# Exploring the Link Between Inflammatory Biomarkers (SII, SIRI, PLR, NLR, LMR) and Migraine in Young and Early Middle‐Aged US Adults: Evidence From NHANES 1999–2004 and Machine Learning Models

**DOI:** 10.1002/brb3.70886

**Published:** 2025-09-21

**Authors:** Guodong Ha, Zixuan Yan, Jiawei Wu, Xun Wang, Jing Hu, Lincheng Duan, Zhengyu Zhao, Dingjun Cai

**Affiliations:** ^1^ Acupuncture and Tuina School Chengdu University of Traditional Chinese Medicine Chengdu Sichuan China

**Keywords:** Inflammatory Biomarkers, LMR, Migraine, NHANES

## Abstract

**Background:**

Migraines are a prevalent neurological condition that significantly impacts quality of life, but the underlying pathophysiology remains unclear. This study aims to explore the relationship between inflammatory biomarkers and migraine prevalence in young and early middle‐aged Americans. The inflammatory biomarkers considered include the Systemic Immune‐Inflammation Index (SII), Systemic Inflammatory Response Index (SIRI), Platelet‐to‐Lymphocyte Ratio (PLR), Neutrophil‐to‐Lymphocyte Ratio (NLR), and Lymphocyte‐to‐Monocyte Ratio (LMR).

**Methods:**

Data from the National Health and Nutrition Examination Survey (NHANES) 1999–2004 were utilized for this investigation. Subgroup analysis, smooth curve fitting, and multivariable logistic regression were employed to evaluate associations. Boruta's algorithm, alongside nine machine learning models, was applied to identify key features. SHapley Additive Explanations (SHAP) values were used to interpret the leading models and highlight influential features.

**Results:**

The study revealed no significant differences in SII, SIRI, NLR, or PLR between individuals with and without migraines. However, a significantly higher LMR was observed in individuals with migraines (mean difference: 0.37, *p *< 0.001). Multivariable logistic regression analysis demonstrated a strong positive correlation between LMR and migraine risk across multiple models (OR = 1.51, 95% CI: 1.14–2.00, *p *= 0.009). No significant associations were found for the other inflammatory biomarkers. Subgroup analyses further confirmed that the positive correlation between LMR and migraine risk remained consistent across different strata. Threshold effect analysis revealed a stable linear relationship between LMR and migraine risk up to a value of 1.61. Among the nine machine learning models, the LightGBM model exhibited the highest AUROC (0.9198), recall (93.3%), *F*1‐score (0.896), and MCC (0.702).

**Conclusions:**

LMR may serve as a potential biomarker for assessing migraine risk, offering support for early diagnosis and personalized intervention strategies.

## Graphical abstract

A strong association was found between LMR and migraine risk, supporting early risk assessment and intervention strategies

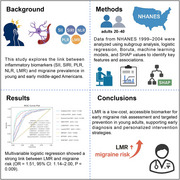

**Abbreviations**
BMI: body mass index
CGRP: calcitonin gene‐related peptide
CRP: C‐reactive protein
CSD: cortical spreading depression
eGFR: estimated glomerular filtration rate
GBDT: Gradient Boosting Decision Tree
LMR: Lymphocyte‐to‐Monocyte Ratio
NLR: Neutrophil‐to‐Lymphocyte Ratio
PIR: poverty‐to‐income ratio
PLR: Platelet‐to‐Lymphocyte Ratio
SHAP: Shapley Additive Explanations
SII: Systemic Immune‐Inflammation Index
SIRI: Systemic Inflammatory Response Index

## 1 Introduction

Migraine, a neurovascular disorder characterized by recurrent moderate to severe headaches, is among the leading causes of disability worldwide, affecting approximately 20% of the population (GBD 2019 Diseases and Injuries Collaborators 2020). It is the leading cause of disability among individuals under the age of 50, with a marked gender disparity: a prevalence of 21% in women compared to 10.7% in men (Burch et al. 2021). Although migraine can occur at any age, its incidence gradually increases from adolescence and peaks in adults aged 20–40 (Riggins and Ehrlich 2022; Fan et al. 2023). The disease burden in this population is particularly substantial, as migraine can profoundly impair daily functioning, occupational productivity, and mental well‐being (Burch et al. 2018). From a socioeconomic perspective, studies indicate that individuals with migraine lose an average of 13.7 productive days annually (Munakata et al. 2009). From a pathophysiological standpoint, approximately 3% of patients progress to chronic migraine, defined as ≥ 15 headache days per month (Messina et al. 2022). This progression is associated with white matter microstructural abnormalities and central sensitization, both of which positively correlate with attack frequency (Messina et al. 2022). These findings suggest that neuroinflammation may play a pivotal role in the chronification of migraine. Therefore, investigating the underlying mechanisms of migraine and optimizing treatment strategies are of both scientific significance and public health importance, necessitating effective interventions to mitigate its global health impact.

Accumulating evidence identifies neuroimmune activation of the trigeminovascular axis as a central driver of migraine pathophysiology, encompassing both episodic and chronic forms. Acute attacks are characterized by elevated IL‐6 and TNF‐α, microglial activation, and subsequent CGRP release, collectively promoting central sensitization and pain hypersensitivity (Perini et al. 2005;Zhang et al. 2023). To capture these complex immunovascular dynamics, composite indices derived from routine blood counts—SII, NLR, and PLR—integrate neutrophil, platelet, and lymphocyte parameters, reflecting systemic immune activation, coagulative shifts, and vascular inflammation in a cost‐effective, widely available manner (Mangoni and Zinellu 2024). Their diagnostic and prognostic value has been demonstrated across diverse settings: SII and NLR track surgical invasiveness and systemic inflammation in orthopedic trauma; NLR and PLR discriminate periprosthetic joint infection from aseptic failure; and all three indices predict cerebrovascular risk. Such cross‐disciplinary evidence supports their application to migraine research, where inflammation–vascular interactions remain incompletely understood (Moldovan 2024a;2024b;Fest et al. 2018). However, existing studies have three major limitations: (1) Most studies focus on single indices, without systematic comparisons of multiple inflammatory biomarkers. (2) Study sample sizes are often small (*n* < 2000), which limits the generalizability and representativeness of the findings. (3) There is inadequate control for sample selection bias and the confounding effects of comorbidities on inflammatory biomarkers. Therefore, conducting large‐scale, systematically designed studies would help clarify the role of these inflammatory biomarkers in both migraine pathogenesis and clinical evaluation.

This study examines the potential associations between migraine and several inflammatory biomarkers—SII, SIRI, NLR, PLR, and LMR—using a representative sample from the NHANES. It aims to shed new light on the immunoinflammatory mechanisms underlying migraine risk and to provide a robust theoretical and data‐driven foundation for early diagnosis, risk assessment, and personalized treatment strategies.

## 2 Methods

### 2.1 Study Population

The NHANES is a continuous, nationally representative cross‐sectional survey that evaluates the nutritional status of the American population. The foundation of public health surveillance, written informed consent was obtained from participants, and all study protocols were approved by the National Center for Health Statistics Ethics Review Board (https://www.cdc.gov/nchs/nhanes/irba98.htm). NHANES employs a stratified, multistage probability sampling design, a method thoroughly validated in epidemiologic studies, to ensure its data are statistically representative. The survey collects comprehensive information spanning demographics, socioeconomic status, dietary habits, and health‐related parameters, with detailed sampling methodologies available on the NHANES website (https://www.cdc.gov/nchs/index.htm).

**FIGURE 1:**
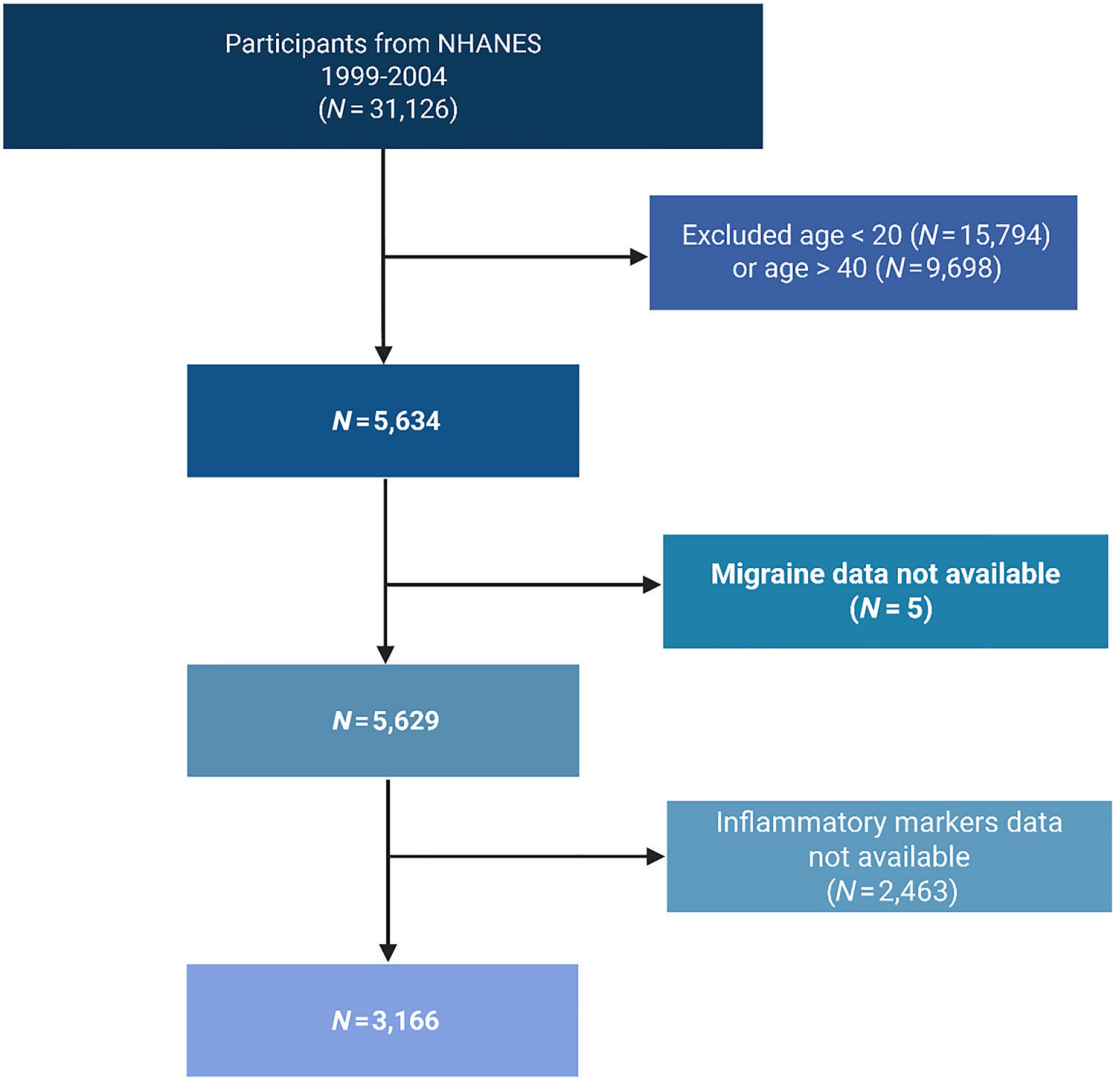
Flowchart of participant selection. (Created in https://BioRender.com).

For the present study, data were initially combined from four successive NHANES cycles (1999–2000, 2001–2002, and 2003–2004), resulting in an initial sample of 31,126 participants. To enhance internal validity, we applied the following sequential exclusion criteria: (1) age outside the range of 20–40 years (*n* = 23,957 excluded); (2) missing migraine‐related data (*n* = 5 excluded); and (3) absence of inflammatory marker measurements (*n* = 2463 excluded). Consequently, the final analytic cohort comprised 3166 participants (Figure 1).

### 2.2 Assessment of Severe Headache or Migraine

A structured pain questionnaire, consistent with established NHANES protocols, was employed to ascertain migraine status (Zhuang et al. 2024). Participants who positively responded to the inquiry regarding experiencing a severe headache or migraine within the past three months were classified as having the condition.

### 2.3 Systemic Immune‐Inflammatory Biomarkers

This study employed biomarkers from complete blood counts, specifically the SII, SIRI, NLR, PLR, and LMR, which have been thoroughly validated as predictors for risk stratification and prognostic assessment in multiple pathologies (Shoji et al. 2020;Ke et al. 2023;J. Li et al. 2023;Wang et al. 2022;M. Li et al. 2021). Laboratory measurements were sourced from NHANES and processed in accordance with the stringent quality control protocols specified in the NHANES Laboratory Procedure Manual (https://www.cdc.gov/nchs/nhanes/biospecimens/serum_plasma_urine.htm). This study assessed the relationship between inflammatory indices and the risk of migraine. The following formulas were used:

- *SII* = (platelet count × neutrophil count)/lymphocyte count
- *SIRI* = (neutrophil count × monocyte count)/lymphocyte count
- *NLR* = neutrophil count/lymphocyte count
- *PLR* = platelet count/lymphocyte count
- *LMR* = lymphocyte count/monocyte count

### 2.4 Covariates

A range of demographic and clinical attributes, such as gender, age, ethnicity, educational attainment, marital status, body mass index (BMI), poverty‐to‐income ratio (PIR), C‐reactive protein (CRP) levels, and estimated glomerular filtration rate (eGFR), were gathered through standardized questionnaires to ascertain potential confounders. Information on educational levels, marital status, smoking status, alcohol consumption, diabetes, hypertension, coronary heart disease, and stroke was obtained via standardized self‐administered questionnaires. Drinking status and smoking status were defined according to questionnaire responses. Participants were classified as having a positive drinking status if they answered “yes” to the question “Had at least 12 alcohol drinks in your lifetime?” and as having a positive smoking status if they answered “yes” to the question “Smoked at least 100 cigarettes in your lifetime?” The CKD–EPI equation was employed to determine eGFR, and laboratory assessments of CRP were conducted (Levey et al. 2009). Furthermore, covariates for the cross‐sectional analysis were selected based on a directed acyclic graph (DAG). After variable selection, sex, age, race, education levels, marital status, BMI, PIR, drinking status, and smoking status were ultimately retained as key covariates in the cross‐sectional analysis. In contrast, all available variables were retained in the predictive modeling. Detailed information was provided in Supporting Information S1.

### 2.5 Statistical Analysis

**FIGURE 2:**
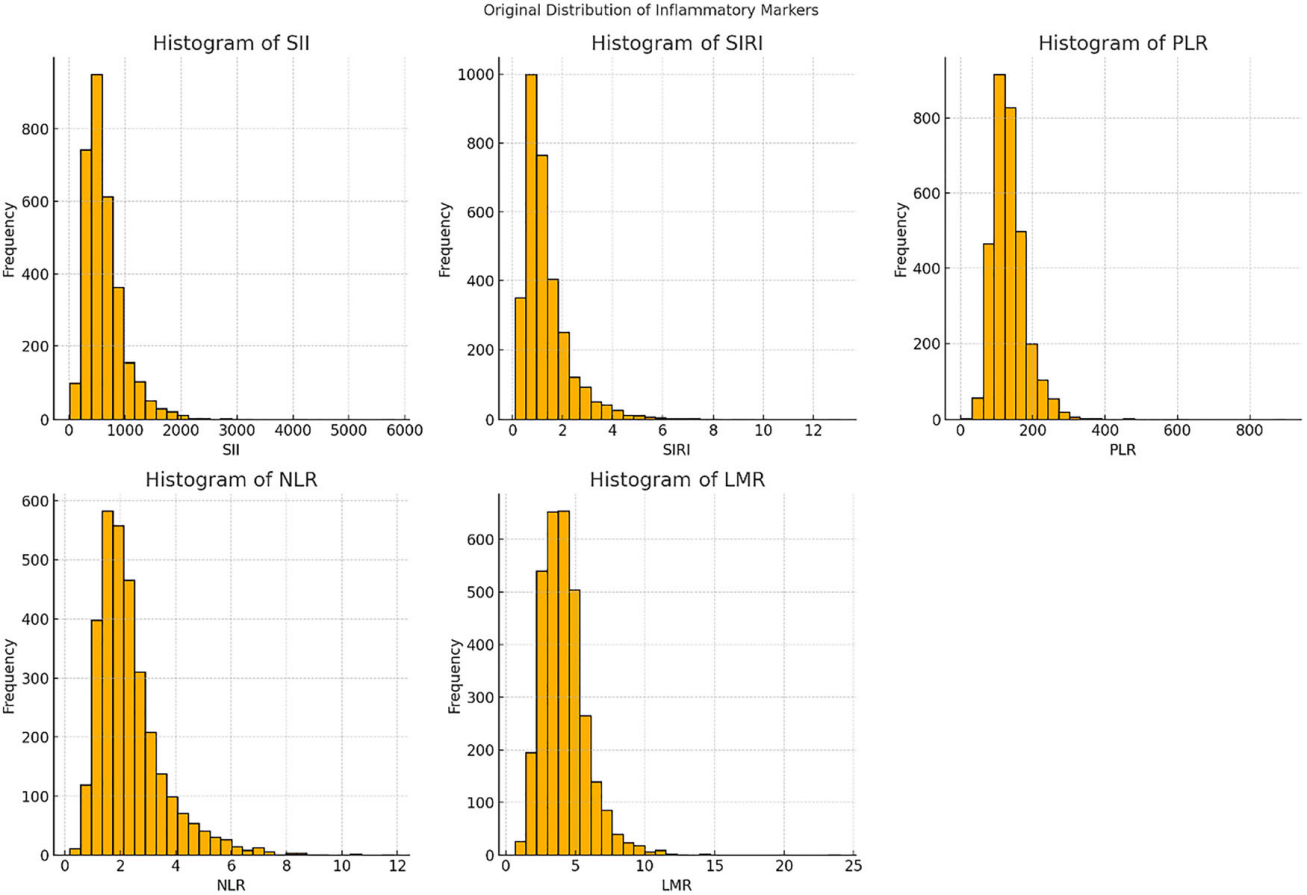
Histograms of SII, SIRI, NLR, PLR, and LMR before and after logarithmic transformation.

**FIGURE 3:**
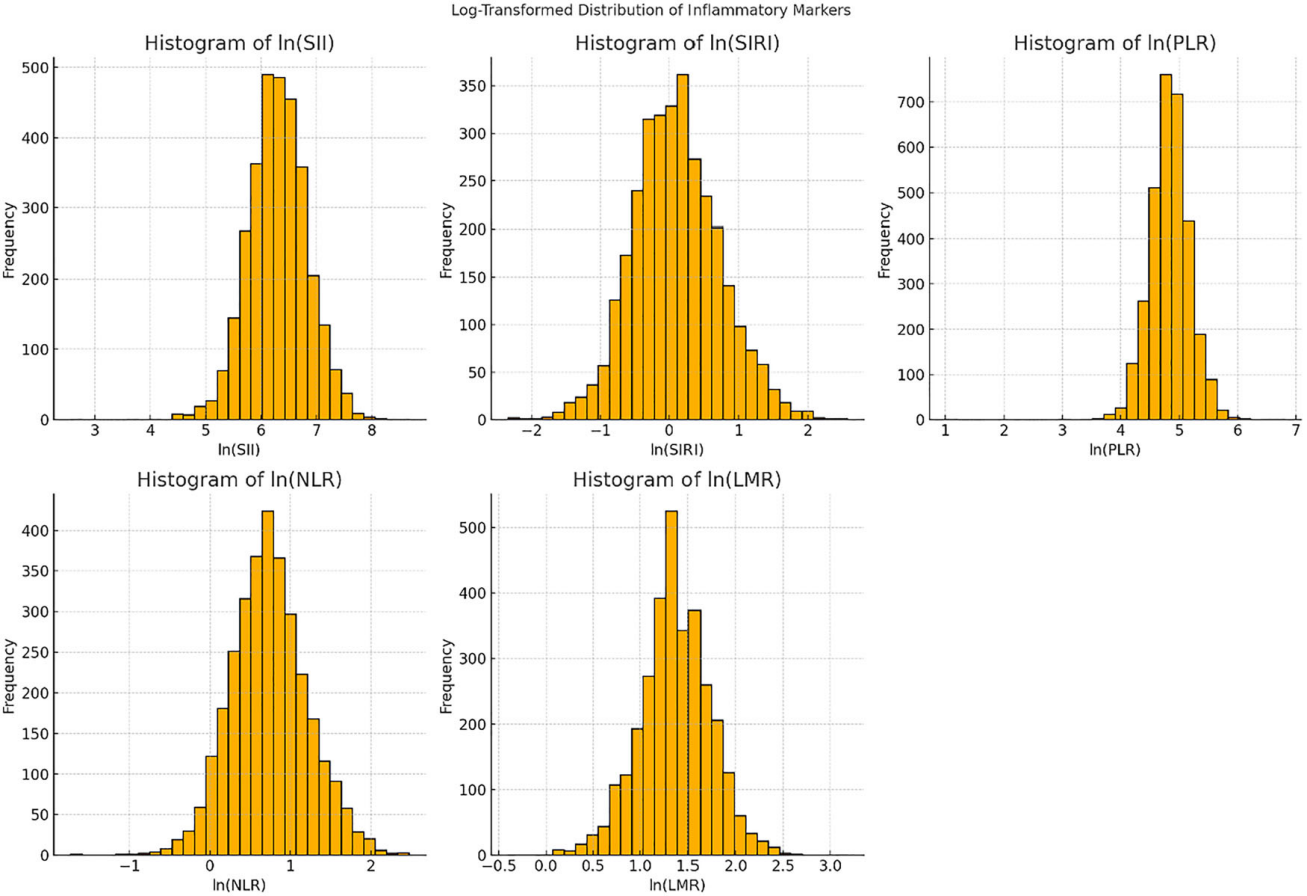
Histograms of SII, SIRI, NLR, PLR, and LMR after logarithmic transformation.

The intricate sample strategy and associated weights were taken into consideration in the analysis. While categorical data are provided as percentages, continuous variables are shown as mean ± standard deviation. Weighted Student's *t*‐tests were employed for continuous data, whereas weighted chi‐square tests were utilized for categorical variables in group comparisons. Variables with a missing rate exceeding 20% (such as alcohol consumption) were excluded from the analysis. Multiple imputation was performed to address the remaining missing data (Muntner et al. 2020). Detailed information was provided in Supporting Information S1. The normality of continuous variables was assessed using the Shapiro–Francia normality test, which is suitable for large sample sizes and sensitive to deviations from normality (Tao et al. 2006;Royston 1993). In addition, histogram plots were visually inspected to confirm the distribution pattern. Variables showing skewed distributions, including SII, SIRI, NLR, PLR, and LMR, were natural log‐transformed to approximate a normal distribution before statistical analyses. Given the skewed distributions of SII, SIRI, NLR, PLR, and LMR, a natural logarithm transformation was performed to approximate normality (Figures 2 and 3 and Supplementary File), after which these indices were stratified into quartiles (Q1–Q4).

We used weighted logistic regression models to examine the association between migraine risk and inflammatory biomarkers, particularly SII, SIRI, NLR, PLR, and LMR. No variables were used in the unadjusted model (Model 1). While Model 2 accounted for age, sex, and race, Model 3 addressed a wide range of confounders, such as sex, age, race, education levels, marital status, BMI, PIR, and smoking status. We also conducted interaction and subgroup analyses. To explore potential nonlinear associations between these biomarkers and migraine risk, smooth curve fitting was applied, and likelihood ratio tests were utilized to assess nonlinearity.

Subsequently, machine learning was applied to evaluate the predictive ability of inflammatory biomarkers for migraine. The Boruta algorithm was used for covariate selection, and SMOTE under‐sampling was employed to balance the dataset. Nine machine learning algorithms—including Decision Tree, Gradient Boosting Decision Tree (GBDT), AdaBoost, LightGBM, Logistic Regression, Random Forest, Naïve Bayes, CatBoost, and XGBoost—were used to construct predictive models for migraine occurrence. The dataset was randomly split into a training set (70%) and a testing set (30%) to ensure model generalizability. A 10‐fold cross‐validation was performed on the training set to optimize hyperparameters and reduce overfitting. To enhance interpretability, SHAP was used to quantify the contribution of each feature.

All statistical analyses were performed using EmpowerStats (http://www.empowerstats.com), R (http://www.R‐project.org), Python (version 3.9.12), and DecisionLinnc1.0 software (Team DC 2023), with a two‐sided *p *< 0.05 deemed statistically significant.

## 3 Results

### 3.1 Characteristics of the Study Population

This survey represented 52,454,905 participants, of whom 13,836,623 suffered from migraine. Table 1 summarizes the baseline characteristics of young and early middle‐aged adults from NHANES (1999–2004). The average age in the non‐migraine and migraine groups was 30.26 ± (6.15) and 30.72 ± (6.06) years, respectively, while the mean BMI was 27.05 ± (6.08) kg/m^2^ in the non‐migraine group compared to 28.69 ± (7.55) kg/m^2^ in the migraine group. Participants with migraine were more frequently female, had higher education level, were overweight, had lower PIR, had increased CRP and eGFR levels, and had no hypertension, diabetes, or stroke, compared to those without migraine (all *p *< 0.05). The migraine group exhibited a notably elevated mean LMR value in comparison to the non‐migraine group (*p *< 0.05). However, no significant differences were found between the groups for SII, SIRI, NLR, or PLR (all *p *> 0.05).

**TABLE 1:** Baseline characteristics from NHANES 1999–2004 among young and early middle‐aged Americans.

Characteristic	*N* ^a^	Overall *(N *= 52,454,905^b^)	Without migraine *(N* = 38,618,282^b^)	With migraine *(N* = 13,836,623^b^)	*p* value^c^
Sex	3166				< 0.001***
Male		1439 (50%)	1195 (57%)	244 (31%)	
Female		1727 (50%)	1119 (43%)	608 (69%)	
Age	3166	30.38 ± (6.13)	30.26 ± (6.15)	30.72 ± (6.06)	0.113
Race	3166				0.568
Non‐Hispanic White		1425 (65%)	1057 (66%)	368 (64%)	
Non‐Hispanic Black		665 (12%)	473 (12%)	192 (14%)	
Mexican American		779 (11%)	563 (11%)	216 (11%)	
Other race—including multi‐racial		108 (4.2%)	85 (4.4%)	23 (3.7%)	
Other Hispanic		189 (7.3%)	136 (7.1%)	53 (7.8%)	
Education level	3166				0.003**
Less than ninth grade		221 (3.9%)	146 (3.5%)	75 (5.3%)	
9–11th grade (includes 12th grade with no diploma)		596 (14%)	422 (14%)	174 (17%)	
High school grade/GED or equivalent		801 (26%)	583 (26%)	218 (25%)	
Some colleges or AA degree		932 (33%)	675 (32%)	257 (35%)	
College graduate or above		616 (23%)	488 (25%)	128 (18%)	
Marital status	3166				0.464
Married		1496 (48%)	1104 (48%)	392 (46%)	
Other		1670 (52%)	1210 (52%)	460 (54%)	
PIR	3166	2.70 ± (1.58)	2.80 ± (1.58)	2.42 ± (1.54)	< 0.001***
BMI	3166	27.48 ± (6.54)	27.05 ± (6.08)	28.69 ± (7.55)	< 0.001***
Smoking status	3166				0.055
Yes		1295 (45%)	919 (44%)	376 (48%)	
No		1871 (55%)	1395 (56%)	476 (52%)	
Hypertension	3166				< 0.001***
Yes		344 (11%)	211 (9.5%)	133 (15%)	
No		2822 (89%)	2103 (90%)	719 (85%)	
Diabetes	3166				0.009**
Yes		57 (1.8%)	30 (1.2%)	27 (3.2%)	
No		3098 (98%)	2277 (98%)	821 (96%)	
Borderline		11 (0.5%)	7 (0.4%)	4 (0.8%)	
Coronary heart disease	3166				0.056
Yes		6 (0.2%)	1 (< 0.1%)	5 (0.5%)	
No		3160 (100%)	2313 (100%)	847 (99%)	
Stroke	3166				0.019*
Yes		13 (0.5%)	6 (0.3%)	7 (1.1%)	
No		3153 (99%)	2308 (100%)	845 (99%)	
CRP	3166	0.37 ± (0.73)	0.35 ± (0.76)	0.43 ± (0.65)	< 0.001***
eGFR	3166	118.49 ± (19.10)	117.25 ± (19.37)	121.95 ± (17.88)	< 0.001***
SII	3166	600.96 ± (346.29)	594.68 ± (331.11)	618.49 ± (385.18)	0.080
SIRI	3166	1.28 ± (0.89)	1.29 ± (0.89)	1.24 ± (0.90)	0.119
PLR	3166	133.31 ± (48.31)	134.08 ± (48.32)	131.17 ± (48.25)	0.180
NLR	3166	2.23 ± (1.07)	2.24 ± (1.06)	2.21 ± (1.11)	0.308
LMR	3166	4.18 ± (1.57)	4.08 ± (1.52)	4.45 ± (1.68)	< 0.001***

*Note*: Continuous variables were expressed as weighted mean (standard deviation, SD), and categorical variables were expressed as number (weighted proportion, %).

^a^ *N* not missing (unweighted).

^b^ *n* (unweighted) (%); mean ± (SD).

^c^ Pearson's *χ* ^2^: Rao and Scott adjustment; design‐based Kruskal–Wallis test.

Abbreviations: BMI, body mass index; LMR, lymphocyte‐monocyte ratio; NLR, neutrophil–lymphocyte ratio; PLR, platelet–lymphocyte ratio; SII, systemic immune inflammation index; SIRI, systemic inflammation response index.

Given the skewed distribution of these inflammatory indicators, SII, SIRI, PLR, NLR, and LMR were ln‐transformed before data analysis.

the asterisk symbols (***) denote statistical significance as follows:***: p <0.001

### 3.2 Association Between Inflammatory Biomarkers and Migraine Risk

Table 2 details the associations between various inflammatory biomarkers and the risk of migraine. We constructed three weighted logistic regression models with progressive adjustments for potential confounders to assess the relationships of SII, SIRI, NLR, PLR, and LMR with migraine risk. In the unadjusted model (Model 1), the natural log‐transformed LMR exhibited a robust positive association with migraine prevalence (OR = 2.01, 95% CI: 1.54–2.60, *p *< 0.001). This association remained statistically significant after adjustment for age, sex, and race in Model 2 (OR = 1.51, 95% CI: 1.14–2.00, *p *= 0.009) and persisted with further adjustment for additional confounders in Model 3 (OR = 1.40, 95% CI: 1.04–1.86, *p *= 0.041). In contrast, Ln (SII), Ln (SIRI), Ln (PLR), and Ln (NLR) did not show significant associations with migraine risk in Model 1; similar patterns were observed in Models 2 and 3 (all *p *> 0.05). Notably, in Model 2 (adjusted for sex, age, and race), Ln (PLR) Q4 showed lower odds of migraine versus Q1 (OR = 0.72, 95% CI: 0.55–0.95). Given the compositional nature of these indices (PLR = platelets/lymphocytes; LMR = lymphocytes/monocytes), opposite directions between PLR and LMR can occur because lymphocyte counts enter the two log‐ratios with opposite signs.

**TABLE 2:** Associations between inflammatory biomarkers and migraine.

Exposures	Model 1 OR (95% CI)	*p* value	Model 2 OR (95% CI)	*p* value	Model 3 OR (95% CI)	*p* value
**Ln (SII)**	1.13 (0.98, 1.31)	0.115	0.99 (0.84, 1.16)	0.867	0.91 (0.76, 1.09)	0.316
Q1	Reference		Reference		Reference	
Q2	1.21 (1.04, 1.40)	0.019*	1.17 (1.00, 1.37)	0.058	1.12 (0.94, 1.32)	0.223
Q3	1.43 (1.13, 1.80)	0.006**	1.27 (0.99, 1.64)	0.079	1.19 (0.91, 1.54)	0.226
Q4	1.20 (0.97, 1.48)	0.102	1.00 (0.80, 1.24)	0.996	0.92 (0.74, 1.14)	0.472
*p* for trend		0.053		0.830		0.634
Ln (SIRI)	0.89 (0.77, 1.04)	0.153	0.94 (0.80, 1.11)	0.494	0.88 (0.74, 1.04)	0.157
Q1	Reference		Reference		Reference	
Q2	0.98 (0.76, 1.28)	0.894	1.11 (0.84, 1.47)	0.471	1.03 (0.77, 1.38)	0.834
Q3	0.92 (0.73, 1.15)	0.469	1.07 (0.83, 1.38)	0.627	1.00 (0.77, 1.30)	0.985
Q4	0.87 (0.65, 1.16)	0.343	0.94 (0.70, 1.26)	0.688	0.83 (0.61, 1.13)	0.267
*p* for trend		0.197		0.541		0.180
Ln (PLR)	0.84 (0.65, 1.10)	0.223	0.74 (0.56, 0.99)	0.051	0.83 (0.60, 1.13)	0.248
Q1	Reference		Reference		Reference	
Q2	0.93 (0.67, 1.29)	0.674	0.86 (0.62, 1.21)	0.395	0.91 (0.65, 1.26)	0.573
Q3	1.04 (0.81, 1.32)	0.784	0.94 (0.73, 1.20)	0.606	1.01 (0.79, 1.28)	0.955
Q4	0.81 (0.62, 1.05)	0.123	0.72 (0.55, 0.95)	0.032*	0.79 (0.60, 1.05)	0.137
*p* for trend		0.230		0.061		0.232
Ln (NLR)	0.90 (0.73, 1.10)	0.308	0.84 (0.65, 1.07)	0.174	0.82 (0.63, 1.07)	0.162
Q1	Reference		Reference		Reference	
Q2	0.96 (0.77, 1.19)	0.728	0.99 (0.77, 1.27)	0.923	1.00 (0.77, 1.29)	0.994
Q3	0.90 (0.71, 1.15)	0.407	0.90 (0.69, 1.19)	0.468	0.87 (0.66, 1.16)	0.377
Q4	0.88 (0.67, 1.16)	0.381	0.81 (0.58, 1.13)	0.233	0.81 (0.58, 1.14)	0.248
*p* for trend		0.319		0.176		0.167
Ln (LMR)	2.01 (1.54, 2.60)	< 0.001***	1.51 (1.14, 2.00)	0.009**	1.40 (1.04, 1.86)	0.041*
Q1	Reference		Reference		Reference	
Q2	1.79 (1.24, 2.57)	0.004**	1.74 (1.23, 2.47)	0.005**	1.74 (1.24, 2.45)	0.008**
Q3	1.69 (1.27, 2.27)	0.002**	1.48 (1.10, 2.01)	0.018*	1.46 (1.07, 2.00)	0.035*
Q4	2.25 (1.63, 3.09)	< 0.001***	1.76 (1.28, 2.43) 0.0025	0.003**	1.66 (1.18, 2.32)	0.013*
*p* for trend		< 0.001***		0.008**		**0.035**

*Note*: Model 1 was not adjusted for any covariates. Model 2 was adjusted for age, gender, and race. Model 3 was adjusted for all covariates based on Model 1.

Abbreviations: CI, confidence interval; LMR, lymphocyte‐to‐monocyte ratio; NLR, neutrophil‐to‐lymphocyte ratio; OR, odds ratio; PLR, platelet‐to‐lymphocyte ratio; Q, quartile; SII, systemic immune‐inflammation index; SIRI, systemic inflammation response index.

the asterisk symbols (***) denote statistical significance as follows: *: p <0.05; **: p < 0.01; ***: p < 0.001

### 3.3 Subgroup Analyses

Subgroup analyses indicated that the positive correlation between LMR and migraine risk remained consistent across various strata, including sex, race, education level, marital status, and smoking status (all *p* for interaction > 0.05) (Tables 3, 4, 5, 6, 7).

**TABLE 3:** The relationship between Ln (SII) and migraine in different subgroups.

Characteristic	OR (95% CI)	*p* for interaction*
Stratified by sex		0.681
Male	0.87 (0.63, 1.19)	
Female	0.94 (0.76, 1.16)	
Stratified by race		0.062
Mexican American	0.92 (0.72, 1.17)	
Other Hispanic	0.91 (0.63, 1.32)	
Non‐Hispanic White	1.00 (0.67, 1.50)	
Non‐Hispanic Black	0.32 (0.14, 0.69)	
Other race—including multi‐racial	1.22 (0.63, 2.38)	
Stratified by education level		0.737
Less than ninth grade	0.94 (0.52, 1.69)	
9–11th grade (includes 12th grade with no diploma)	0.62 (0.35, 1.12)	
High school grade/GED or equivalent	0.99 (0.67, 1.45)	
Some colleges or AA degree	0.98 (0.69, 1.38)	
College graduate or above	0.96 (0.59, 1.58)	
Stratified by marital status		0.585
Married	0.98 (0.73, 1.30)	
Other	0.86 (0.63, 1.16)	
Stratified by smoking status		0.941
Yes	0.90 (0.63, 1.28)	
No	0.92 (0.72, 1.17)	

**TABLE 4:** The relationship between Ln (SIRI) and migraine in different subgroups.

Characteristic	OR (95% CI)	*p* for interaction
Stratified by sex		0.481
Male	0.83 (0.69, 1.01)	
Female	0.90 (0.74, 1.10)	
Stratified by race		0.775
Mexican American	0.87 (0.69, 1.08)	
Other Hispanic	0.87 (0.58, 1.30)	
Non‐Hispanic White	0.88 (0.61, 1.25)	
Non‐Hispanic Black	0.54 (0.15, 1.94)	
Other race—including multi‐racial	1.21 (0.66, 2.24)	
Stratified by education level		0.752
Less than ninth grade	0.58 (0.26, 1.33)	
9–11th grade (includes 12th grade with no diploma)	0.87 (0.52, 1.45)	
High school grade/GED or equivalent	0.94 (0.66, 1.34)	
Some colleges or AA degree	0.95 (0.72, 1.25)	
College graduate or above	0.77 (0.50, 1.17)	
Stratified by marital status		0.814
Married	0.90 (0.67, 1.21)	
Other	0.86 (0.67, 1.09)	
Stratified by smoking status		0.725
Yes	0.91 (0.68, 1.23)	
No	0.85 (0.67, 1.08)	

**TABLE 5:** The relationship between Ln (PLR) and migraine in different subgroups.

Characteristic	OR (95% CI)	*p* for interaction
Stratified by sex		0.556
Male	0.75 (0.48, 1.18)	
Female	0.89 (0.61, 1.30)	
Stratified by race		0.202
Mexican American	0.86 (0.58, 1.27)	
Other Hispanic	0.79 (0.46, 1.34)	
Non‐Hispanic White	1.16 (0.59, 2.31)	
Non‐Hispanic Black	0.20 (0.04, 0.91)	
Other race—including multi‐racial	0.84 (0.27, 2.60)	
Stratified by education level		0.030
Less than ninth grade	1.24 (0.37, 4.19)	
9‐11th grade (includes 12th grade with no diploma)	0.38 (0.23, 0.62)	
High school grade/GED or equivalent	0.96 (0.57, 1.62)	
Some colleges or AA degree	0.74 (0.36, 1.50)	
College graduate or above	1.37 (0.62, 3.03)	
Stratified by marital status		0.729
Married	0.87 (0.59, 1.27)	
Other	0.79 (0.50, 1.24)	
Stratified by smoking status		0.255
Yes	0.68 (0.40, 1.16)	
No	0.97 (0.70, 1.35)	

**TABLE 6:** The relationship between Ln (NLR) and migraine in different subgroups.

Characteristic	OR (95% CI)	*p* for interaction
Stratified by sex		0.444
Male	0.74 (0.49, 1.12)	
Female	0.86 (0.64, 1.16)	
Stratified by race		0.478
Mexican American	0.76 (0.53, 1.09)	
Other Hispanic	0.99 (0.65, 1.52)	
Non‐Hispanic White	1.05 (0.70, 1.57)	
Non‐Hispanic Black	0.37 (0.11, 1.22)	
Other race—including multi‐racial	1.17 (0.46, 2.96)	
Stratified by education level		0.910
Less than ninth grade	0.70 (0.34, 1.44)	
9–11th grade (includes 12th grade with no diploma)	0.61 (0.31, 1.20)	
High school grade/GED or equivalent	0.87 (0.54, 1.41)	
Some colleges or AA degree	0.87 (0.59, 1.30)	
College graduate or above	0.87 (0.49, 1.55)	
Stratified by marital status		0.522
Married	0.90 (0.61, 1.32)	
Other	0.75 (0.52, 1.10)	
Stratified by smoking status		0.799
Yes	0.87 (0.55, 1.40)	
No	0.78 (0.58, 1.04)	

**TABLE 7:** The relationship between Ln (LMR) and migraine in different subgroups.

Characteristic	OR (95% CI)	*p* for interaction
Stratified by sex		0.444
Male	1.57 (1.03, 2.39)	
Female	1.30 (0.93, 1.82)	
Stratified by race		0.746
Mexican American	1.43 (0.96, 2.12)	
Other Hispanic	1.47 (0.78, 2.77)	
Non‐Hispanic White	1.23 (0.79, 1.89)	
Non‐Hispanic Black	2.82 (0.71, 11.23)	
Other race—including multi‐racial	0.95 (0.37, 2.43)	
Stratified by education level		0.783
Less than ninth grade	2.54 (0.94, 6.88)	
9–1th grade (includes 12th grade with no diploma)	1.31 (0.68, 2.53)	
High school grade/GED or equivalent	1.18 (0.73, 1.90)	
Some colleges or AA degree	1.45 (0.81, 2.61)	
College graduate or above	1.50 (0.71, 3.14)	
Stratified by marital status		0.830
Married	1.44 (0.97, 2.16)	
Other	1.36 (0.90, 2.05)	
Stratified by smoking status		0.799
Yes	1.44 (0.99, 2.11)	
No	1.35 (0.92, 1.99)	

### 3.4 Smooth Curve Fitting and Threshold Effect Analysis

**FIGURE 4:**
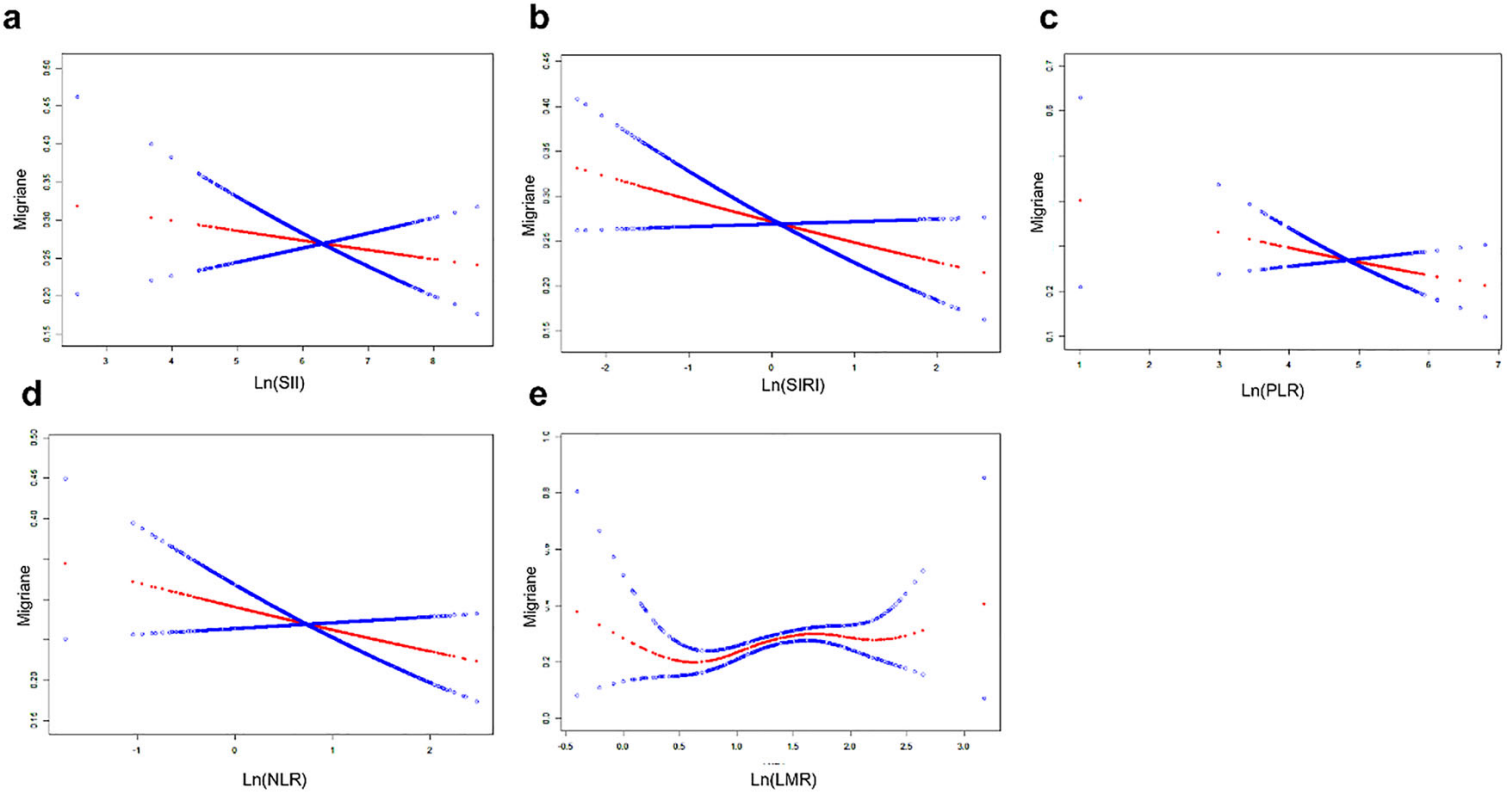
The association between systemic inflammatory biomarkers (ln‐transformed) and migraine.

To further validate the robustness of our findings, we explored the potential nonlinear associations between the systemic inflammatory biomarkers (SII, SIRI, NLR, PLR, and LMR) and migraine risk using smooth curve fitting adjusted for all confounders (Figure 4). In addition, we performed piecewise regression and threshold analyses specifically for LMR (Table 8). The threshold analysis indicated that increases in Ln (LMR) were strongly and positively associated with migraine risk up to an inflection point of 1.61; beyond this threshold, the association lost significance. Nevertheless, the likelihood ratio test (LLR > 0.05), along with the significant linear effect in the initial model (Model I, p < 0.05), suggests that a linear relationship best characterizes the association between LMR and migraine risk.

**TABLE 8:** The threshold effect of LMR (Ln‐transformed) on migraine was analyzed using a two‐stage phased regression model.

	OR (95% CI)	*p* value
Model I		
logistic regression (the standard linear model)	1.40 (1.12, 1.74)	0.003**
Model II		
Inflection point (*K*)	1.61	
< *K*	1.71 (1.25, 2.33)	0.001**
> *K*	0.81 (0.43, 1.53)	0.516
Log likelihood ratio (LLR)		0.069

the asterisk symbols (***) denote statistical significance as follows: **: p < 0.01

The red line represents the smoothing curve fitting between the variables. The blue band indicates the fitted 95% confidence interval.

### 3.5 Machine Learning Analysis

**FIGURE 5:**
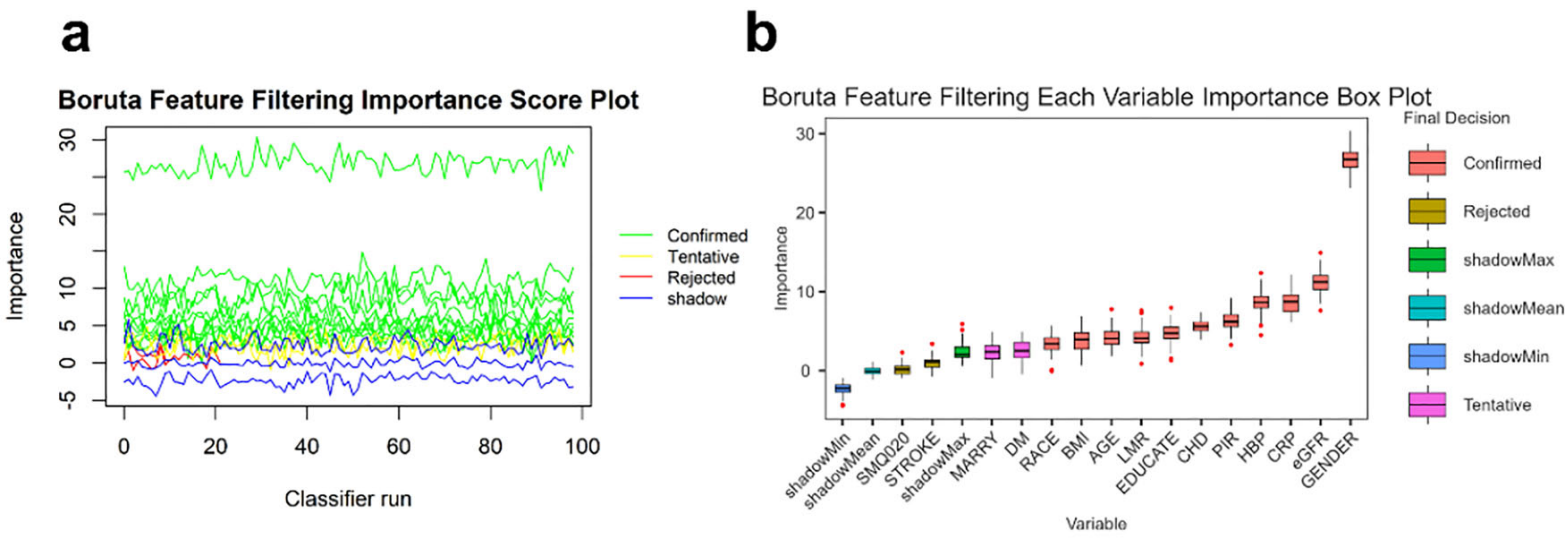
Results on feature selection for LMR based on Boruta's algorithm.

Based on the Boruta feature selection algorithm, 13 variables were confirmed as important predictors, including sex, eGFR, CRP, hypertension, PIR, coronary heart disease, education level, LMR, age, BMI, race, diabetes and marital status, while two variables (smoking status and stroke) were excluded due to insufficient importance (Figure 5).

**FIGURE 6:**
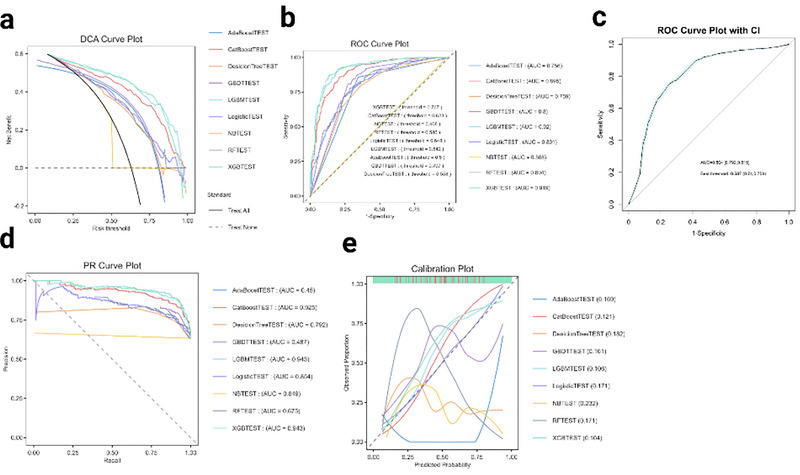
Multi‐model evaluation of test set results on LMR. (a) DCA (Decision Curve Analysis) (b) ROC curve (c) The ROC evaluation curve with confidence intervals (d) The PR (Precision–Recall) curve (e) Calibration curve.

In the test set, the study evaluated the performance of nine machine learning models in predicting migraine risk among early middle‐aged and young adults (Figure 6 and Supplorting Information S2). Considering comprehensive indicators including accuracy, AUROC, *F*1‐score, MCC, PR‐AUC, calibration, and net clinical benefit (DCA), the LightGBM model achieved the best overall performance. It had the highest AUROC (0.9198), recall (93.3%), *F*1‐score (0.896), and MCC (0.702), with the lowest false negative rate (6.7%) and false positive rate (25.6%). The XGBoost model ranked second, with an AUROC of 0.9191 and a slightly higher recall (93.5%), but slightly lower precision and MCC. Based on the above indicators, LightGBM performed best in the test set and was suitable as the core model for subsequent explanatory analysis.

**FIGURE 7:**
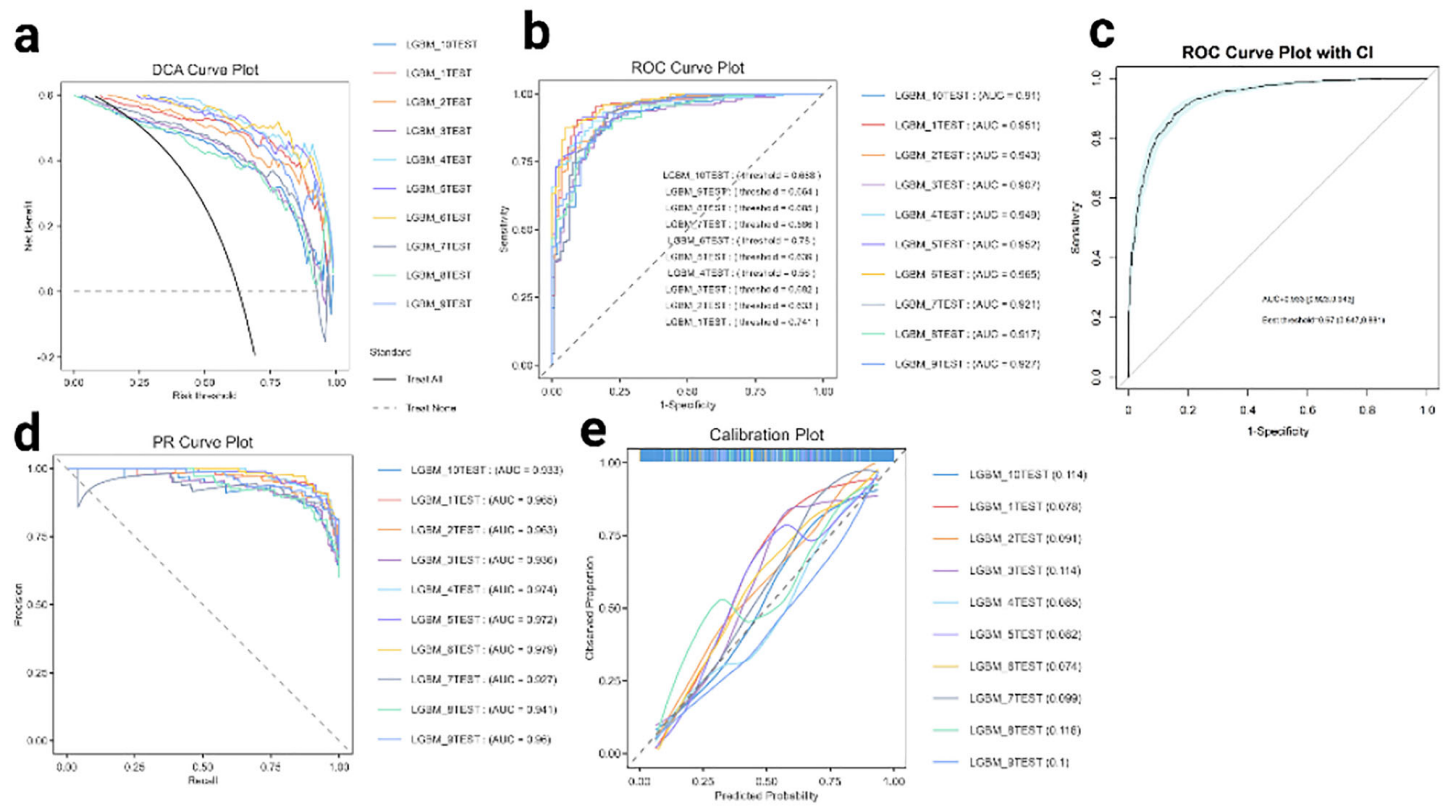
Cross‐validated multi‐model assessment results on LMR. (a) DCA (Decision Curve Analysis) (b) ROC curve (c) The ROC evaluation curve with confidence intervals (d) The PR (Precision–Recall) curve (e) Calibration curve.

In order to verify the generalization ability of the model, a 10‐fold cross‐validation evaluation of the LightGBM model was further performed (Figure 7). In the ROC curve, all LightGBM models exhibited stable and high predictive power, with AUC ranging from 0.907 to 0.979. The cross‐validation results confirmed that the LightGBM model had good stability and generalization ability.

**FIGURE 8:**
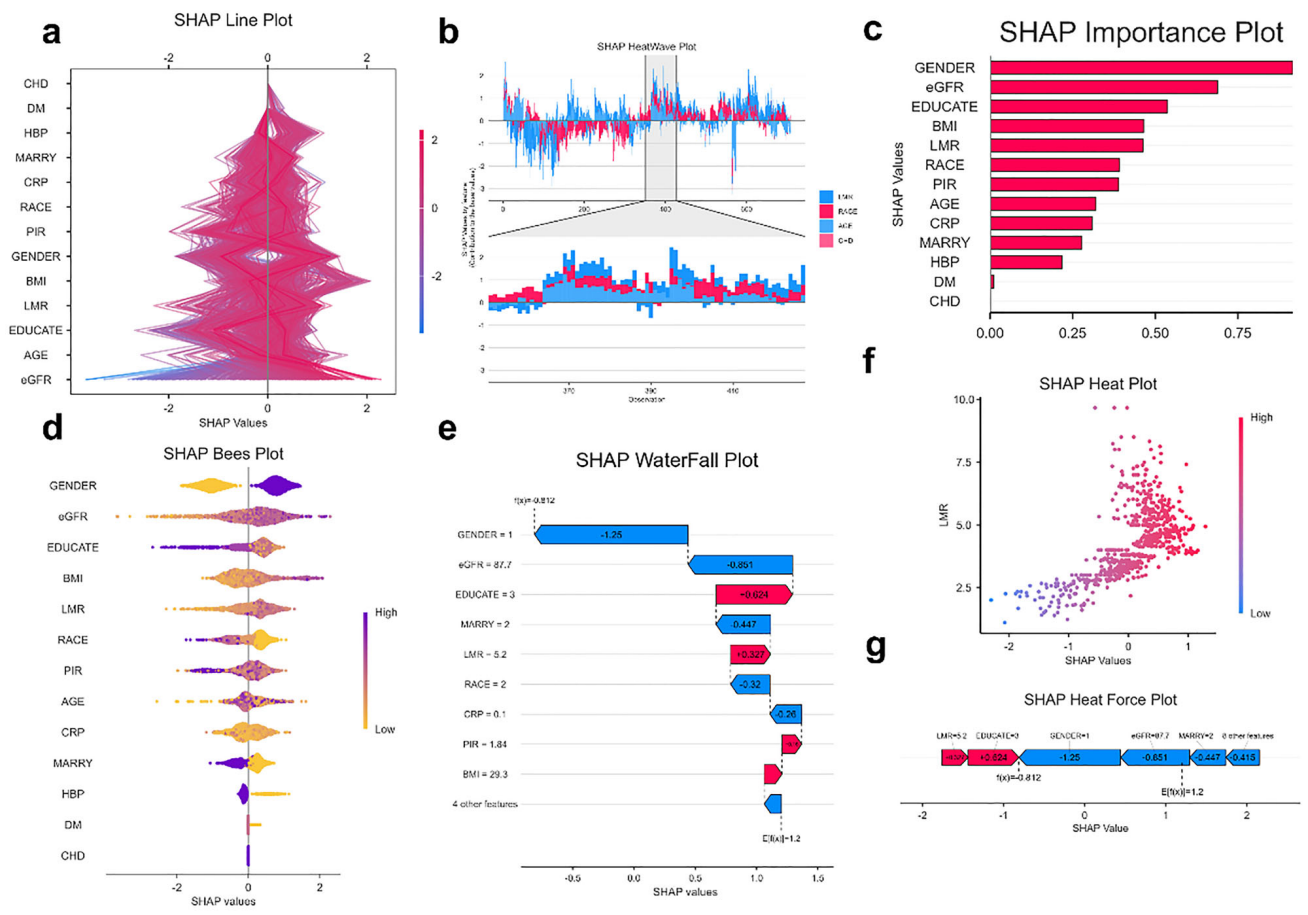
Shapley Additive Explanations (SHAP) analyzes LightGBM models on LMR. (a) SHAP line plot, (b) SHAP heatwave plot, (c) SHAP importance plot, (d) SHAP bees plot, (e) SHAP waterfall plot, (f) SHAP heatmap scatter plot, and (g) SHAP heat force plot.

To further elucidate the model's understanding of the relationship between LMR and migraine, SHAP analysis was conducted on the best‐performing model in the test set—LightGBM (Figure 8).

The SHAP summary bar plot (Figure 8c) revealed that sex, eGFR, education level, and BMI were the most influential features, with LMR ranking fifth, indicating its significant independent contribution to the model's prediction. Both the SHAP line plot (Figure 8a) and bee swarm plot (Figure 8d) demonstrated the bidirectional impact of LMR on model output—higher LMR values were generally associated with an increased risk of migraine.

The SHAP waterfall plot (Figure 8e) illustrated the positive and negative contributions of variables such as LMR, sex, and eGFR in a representative individual, clearly visualizing the specific impact of LMR on the model's decision. The SHAP heatmap (Figure 8f) and force plot (Figure 8g) further confirmed that higher LMR values corresponded to larger positive SHAP values, suggesting that LMR may increase migraine risk by promoting a pro‐inflammatory state.

## 4 Discussion

This study examined the relationship between systemic inflammatory biomarkers (SII, SIRI, NLR, PLR, and LMR) and the risk of migraine to elucidate the potential role of inflammation in migraine pathogenesis. The study demonstrates a significant positive correlation between LMR and migraine prevalence, and LMR exhibits strong predictive performance for identifying migraine in early middle‐aged and young populations. This study is the first to investigate these inflammatory biomarkers of migraine, providing novel theoretical insights and empirical evidence for early diagnosis and personalized management strategies.

The inflammatory indices evaluated here have been widely applied in prognostic assessments across various conditions. For instance, SII and SIRI, which reflect the balance among neutrophils, platelets, and lymphocytes, have been shown to predict outcomes in cardiovascular diseases, cancers, and autoimmune disorders (J. H. Chen et al. 2017;Xie et al. 2018;Jin et al. 2021;C. H. Yang et al. 2025). Similarly, NLR and PLR, by quantifying the interplay between innate immune cells (neutrophils, platelets) and adaptive immune cells (lymphocytes), serve as robust indicators of systemic inflammatory status (Zahorec 2021;Gasparyan et al. 2019). Clinical studies have linked elevated NLR and PLR with increased in‐hospital mortality in acute myocardial infarction, heightened rheumatoid arthritis activity, and greater risk of multiorgan failure in sepsis (Y. Chen et al. 2023;Masoumi et al. 2024;Zinellu and Mangoni 2023;X. Li et al. 2024). LMR is a crucial biomarker for assessing inflammatory status and immune function. In autoimmune diseases, an elevated LMR is associated with an overproduction of pro‐inflammatory cytokines driven by monocytes, possibly indicating the activation of compensatory anti‐inflammatory pathways. In contrast, in cardiovascular diseases, a reduced LMR may reflect immune dysregulation within a predominantly pro‐inflammatory environment (X. Li et al. 2024). These biomarkers provide a theoretical basis for studying the relationship between migraine and systemic inflammation. Our study found a significant association between migraine risk and these biomarkers, suggesting that further research is needed to elucidate their roles in the pathogenesis of migraine.

Our findings indicate that higher LMR is associated with an increased prevalence of migraine. The opposite direction observed for PLR likely reflects differences in the cellular components emphasized by the two indices and the mathematical properties of log‐ratios: ln (PLR) decreases as lymphocyte counts rise, whereas ln (LMR) increases. Consequently, lymphocyte‐driven changes may manifest in opposite directions across these indices. Moreover, adjustment for sex, age, and race—variables correlated with baseline hematologic profiles—may further attenuate quartile contrasts without undermining biological plausibility.

The increase in LMR observed in migraine patients may be driven by several interrelated pathophysiological processes. First, during acute migraine episodes, the activation of the trigeminovascular system leads to the release of neuropeptides such as CGRP and substance P. These mediators not only trigger mast cell degranulation but also facilitate the migration of monocytes toward the meninges, potentially causing a transient reduction in circulating monocytes and a compensatory elevation in LMR (Eftekhari et al. 2013;Edvinsson et al. 2021). Second, alterations in lymphocyte subpopulations—specifically an imbalance between Th17 cells and regulatory T cells observed in chronic migraine—may disrupt cytokine homeostasis, compromise blood–brain barrier integrity, and exacerbate central sensitization, thereby influencing LMR dynamics (L. Yang et al. 2023;Biscetti et al. 2022;2021). Third, bidirectional neuroimmune interactions are implicated; experimental evidence suggests that cortical spreading depression (CSD) can activate microglia and upregulate the TLR4 pathway, promoting the infiltration of peripheral monocytes into the central nervous system and further modulating the LMR (He et al. 2023;Ramachandran et al. 2019;Kaya et al. 2025). Importantly, an elevated LMR may not merely signal an intensified inflammatory state but also reflect the redistribution and activation status of immune cells.

Subgroup analyses showed different patterns of connection between migraine risk and different inflammatory biomarkers. Interestingly, the positive correlation between the prevalence of migraine and the LMR held for a variety of subgroups, including those based on sex, race, education level, marital status, and smoking status (all interaction *p *> 0.05). This consistency lends credence to the idea that LMR may be a reliable and widely used biomarker for migraine risk, as it indicates that these variables do not substantially alter the LMR‐migraine connection. Biologically, LMR reflects the balance between anti‐inflammatory lymphocytes and pro‐inflammatory monocytes—cell types that are central to immune regulation, inflammation, and tissue repair. Given that migraine pathogenesis may involve a state of chronic low‐grade inflammation leading to alterations in immune cell distribution and function, an elevated LMR might directly mirror persistent inflammatory and immune dysregulation in migraine sufferers (Ligthart et al. 2016).

This study identifies LMR as a potential independent biomarker for migraine prevalence in young adults, supporting the neuroimmune hypothesis of migraine pathogenesis, which involves systemic inflammation and immune dysregulation. As an easily accessible, cost‐effective marker derived from routine complete blood counts, LMR shows promise for clinical application in migraine risk stratification, treatment monitoring, and personalized therapy: (1) risk stratification: LMR could facilitate the early identification of individuals at elevated risk for migraines. A low LMR, indicative of a pro‐inflammatory state, correlates with increased susceptibility, particularly in the presence of other risk factors (Arzani et al. 2020). Incorporating LMR into primary care or headache clinic screening protocols could enable timely preventive interventions (e.g., lifestyle modifications, prophylactic pharmacotherapy) for individuals with an LMR below a defined threshold (e.g., the lowest quartile). Integrating LMR with established risk factors (age, sex, BMI, and family history) could enhance migraine risk prediction models, similar to how inflammatory biomarkers are used in cardiovascular risk assessment (Gelaye et al. 2017;Sun et al. 2025). (2) Monitoring treatment response: LMR holds potential as an objective biomarker for tracking therapeutic efficacy. Serial measurements could reflect real‐time responses to anti‐inflammatory or anti‐CGRP therapies (Wijeratne et al. 2025;De Icco et al. 2020). An increase in LMR may signify a positive response, while persistently low levels despite symptomatic improvement could indicate the need for therapy escalation (e.g., dose adjustment, agent switching, or combination therapy). This would aid long‐term management decisions. (3) Personalized intervention strategies: LMR could guide targeted therapeutic approaches based on individual inflammatory profiles. Patients with low LMR (suggesting lymphopenia/monocytosis) may benefit preferentially from immunomodulatory strategies (e.g., cytokine‐targeting biologics such as IL‐1β/IL‐6 inhibitors or microglial modulators) (Mallick et al. 2025;da Costa et al. 2024). Conversely, patients with higher LMR might respond better to neuromodulatory or vascular‐targeted treatments. Personalized lifestyle interventions could also be monitored via follow‐up LMR measurements. Furthermore, integrating LMR with digital health tools (e.g., point‐of‐care devices) could enable real‐time inflammatory profiling and timely intervention to prevent migraine progression. In conclusion, LMR demonstrates significant potential as a predictive and prognostic biomarker in migraine management. Its simplicity, accessibility, and cost‐effectiveness make it a valuable tool for clinical use. By improving risk assessment, treatment monitoring, and personalized therapy, LMR could substantially optimize migraine management strategies and enhance patient outcomes.

This study focuses on the adult population aged 20–40, as epidemiological research indicates that the prevalence and disease burden of migraine increase rapidly after puberty, peaking between the ages of 30 and 44, and then gradually decline with age (Dong et al. 2025). Selecting this age group helps capture “active cases,” improves statistical power, and meets the practical needs of public health interventions. Furthermore, as individuals age, the body gradually enters a chronic inflammatory state, with baseline levels of certain inflammatory biomarkers consistently rising (Tylutka et al. 2024). During the 20–40 age range, the immune system and physiological state of adults are relatively stable, with no significant decline in the immune system and minimal influence from severe chronic diseases or immune dysregulation. This makes it an ideal period for accurately assessing the relationship between migraine and inflammatory biomarkers. Finally, the ages of 20–40 represent the peak of fertility and workforce productivity, where the impact of migraine on work efficiency and quality of life is particularly significant. Therefore, studying migraine characteristics in this population is crucial for workplace health management and early intervention strategies (Dueland et al. 2004).

Despite these encouraging results, it is important to recognize a number of limitations. First, the cross‐sectional design makes it impossible to draw firm conclusions about the causal relationship between elevated LMR and migraine; the lack of temporal data makes it difficult to determine whether changes in LMR precede or result from migraine and limits our understanding of the dynamic patterns of attack frequency and duration. Although internal validation was performed, external validation in an independent cohort is still warranted to enhance the robustness and generalizability of the model. Second, although adjustments were made for several covariates, potential biases in covariate selection and measurement—along with unmeasured lifestyle and environmental factors (e.g., air pollution, psychological stress)—might have influenced the results. Third, while the NHANES data are nationally representative, the relatively small number of migraine cases among adults aged 20–40 may have reduced the precision of our statistical analyses and limited subgroup evaluations based on race, sex, or migraine subtype. Finally, reliance on self‐reported symptoms and medical records for migraine diagnosis may have led to the underestimation of milder or intermittent cases, potentially affecting the reliability of our conclusions. Future research should aim to enlarge the sample size across broader age ranges and diverse ethnic backgrounds and incorporate stricter clinical diagnostic criteria—potentially supplemented by imaging or biomarker assessments—to enhance diagnostic accuracy.

## 5 Conclusion

In summary, our study demonstrates that among adults aged 20–40, LMR is significantly and positively associated with migraine prevalence, whereas other inflammatory biomarkers (SII, SIRI, NLR, and PLR) do not exhibit significant associations. To confirm these results and investigate the pathogenic mechanisms behind the association between migraine and inflammatory biomarkers, more longitudinal and mechanistic studies are necessary.

## Author Contributions

**Guodong Ha**: conceptualization, formal analysis, writing – original draft, writing – review and editing. **Zixuan Yan**: data curation, formal analysis, writing – review and editing, writing – original draft. **Jiawei Wu**: supervision, writing – review and editing. **Xun Wang**: supervision, writing – review and editing. **Jing Hu**: writing – review and editing. **Lincheng Duan**: data curation, writing – review and editing. **Zhengyu Zhao**: writing – review and editing. **Dingjun Cai**: project administration, writing – review and editing.

## Ethics Statement

All participants provided written informed consent, and the study procedures were approved by the National Center for Health Statistics Research Ethics Review Board.

## Conflicts of Interest

The authors declare no conflicts of interest.

## Peer Review

The peer review history for this article is available at https://publons.com/publon/10.1002/brb3.70886.

## Supporting information



Supporting Information: brb370886‐sup‐0001‐SuppMat.xlsx

## Data Availability

The datasets analyzed in this study are publicly available. These can be accessed on the NHANES website. (https://www.cdc.gov/nchs/nhanes/index.htm).
